# The SYGMA programme of phase 3 trials to evaluate the efficacy and safety of budesonide/formoterol given ‘as needed’ in mild asthma: study protocols for two randomised controlled trials

**DOI:** 10.1186/s13063-016-1731-4

**Published:** 2017-01-10

**Authors:** Paul M. O’Byrne, J. Mark FitzGerald, Nanshan Zhong, Eric Bateman, Peter J. Barnes, Christina Keen, Gun Almqvist, Kristine Pemberton, Carin Jorup, Stefan Ivanov, Helen K. Reddel

**Affiliations:** 1 Michael G DeGroote School of Medicine, Faculty of Health Sciences, McMaster University, Hamilton, ON Canada; 2 Institute for Heart and Lung Health, University of British Columbia, Vancouver, BC Canada; 3 State Key Laboratory of Respiratory Diseases, First Affiliated Hospital, Guangzhou Medical University, Guangzhou, China; 4 Department of Medicine, University of Cape Town, Cape Town, South Africa; 5 Airway Disease Section, National Heart and Lung Institute, Imperial College, London, UK; 6 AstraZeneca R&D, Gothenburg, Sweden; 7 AstraZeneca, Alderley Park, Macclesfield, UK; 8 Clinical Management Group, Woolcock Institute of Medical Research, University of Sydney, Sydney, Australia; 9 Firestone Institute of Respiratory Health, St Joseph’s Healthcare and Department of Medicine, McMaster University, 1280 Main Street West, Hamilton, ON L8S 4K1 Canada

**Keywords:** As-needed, Asthma control, Budesonide/formoterol, Exacerbations, Mild asthma, Prn, Rescue inhaler, SYGMA

## Abstract

**Background:**

In many patients with mild asthma, the low frequency of symptoms and the episodic nature of exacerbations make adherence to regular maintenance treatment difficult. This often leads to over-reliance on short-acting β_2_-agonist (SABA) reliever medication and under-treatment of the underlying inflammation, with poor control of asthma symptoms and increased risk of exacerbations. The use of budesonide/formoterol ‘as needed’ in response to symptoms may represent an alternative treatment option for patients with mild asthma.

**Methods/design:**

The SYmbicort Given as needed in Mild Asthma (SYGMA) programme consists of two 52-week, double-blind, randomised, multicentre, parallel-group, phase 3 trials of patients aged 12 years and older with a clinical diagnosis of asthma for at least 6 months, who would qualify for treatment with regular inhaled corticosteroids (ICS). SYGMA1 aims to recruit 3750 patients who will be randomised to placebo twice daily (bid) plus as-needed budesonide/formoterol 160/4.5 μg, placebo bid plus as-needed terbutaline 0.4 mg, or budesonide 200 μg bid plus as-needed terbutaline 0.4 mg. The primary objective is to demonstrate the superiority of as-needed budesonide/formoterol over as-needed terbutaline for asthma control, as measured by well-controlled asthma weeks; a secondary objective is to establish the noninferiority of as-needed budesonide/formoterol versus maintenance budesonide plus as-needed terbutaline using the same outcome measure. SYGMA2 aims to recruit 4114 patients who will be randomised to placebo bid plus as-needed budesonide/formoterol 160/4.5 μg, or budesonide 200 μg bid plus as-needed terbutaline 0.4 mg. The primary objective is to demonstrate the noninferiority of as-needed budesonide/formoterol over budesonide bid plus as-needed terbutaline as measured by the annualised severe exacerbation rate. In both studies, use of all blinded study inhalers will be recorded electronically using Turbuhaler® Usage Monitors.

**Discussion:**

Given the known risks of mild asthma, and known poor adherence with regular inhaled corticosteroids, the results of the SYGMA programme will help to determine the efficacy and safety of as-needed budesonide/formoterol therapy in mild asthma. Patient recruitment is complete, and completion of the phase 3 studies is planned in 2017.

**Trial registration:**

ClinicalTrials.gov identifiers: NCT02149199 SYGMA1 and NCT02224157 SYGMA2. Registered on 16 May 2014 and 19 August 2014, respectively.

**Electronic supplementary material:**

The online version of this article (doi:10.1186/s13063-016-1731-4) contains supplementary material, which is available to authorized users.

## Background

Approximately 50–75% of patients with asthma are considered to have mild disease, but the risks, especially of exacerbations, are often poorly recognised [1]. The long-term goals of asthma management, including mild disease, are to achieve good symptom control and minimise the future risk of exacerbations, fixed airflow limitation and side effects [2]. However, asthma remains uncontrolled in many patients, despite the availability of effective treatment regimens [1, 3].

Although inhaled corticosteroids (ICS) are an effective treatment for asthma, poor adherence to prescribed maintenance therapy results in under-treatment of the underlying inflammation and an increased risk of exacerbations, disease progression and death [4–9]. In addition, over-reliance on short-acting β_2_-agonist (SABA) reliever medication for symptomatic improvement often leads to a delay in the introduction of ICS in patients with mild asthma [10]. Consequently, an alternative approach could be to use an ICS in combination with either a SABA or a rapid- and long-acting β_2_-agonist (LABA) as reliever medication without concurrent maintenance treatment, ensuring that ICS is delivered whenever the patient experiences asthma symptoms, and allowing titration of both ICS, and SABA or LABA, according to patient need.

There is evidence to suggest that a combination of ICS and a rapid-acting β_2_-agonist is effective when given ‘as needed’ in response to asthma symptoms, across all disease severities. In patients with mild asthma, the BEST study showed that the as-needed use of beclometasone dipropionate (BDP) and salbutamol in a single inhaler was noninferior to regular ICS maintenance therapy [11]. For mild-to-moderate asthma, another study has shown that the time to treatment failure was similar when patients received as-needed BDP and salbutamol in separate inhalers compared with a maintenance dose of ICS adjusted at 6-weekly intervals based on physician assessment [12]. Similarly, as-needed budesonide/formoterol was shown to reduce fractional exhaled nitric oxide (FeNO) and improve lung function compared with as-needed formoterol in patients with so-called intermittent asthma and elevated baseline FeNO [13]. In moderate-to-severe asthma, as-needed budesonide/formoterol used in addition to maintenance therapy reduced the rate of severe exacerbations compared with either formoterol or terbutaline reliever therapy, as shown in the Symbicort SMART™ programme [14]. Consequently, there is a need for large-scale randomised clinical trials to further assess this as-needed approach in patients with mild asthma, as highlighted in recent reviews [15, 16].

The SYmbicort Given as needed in Mild Asthma (SYGMA) programme aims to evaluate the efficacy and safety of as-needed budesonide/formoterol in patients with mild asthma. Here, we describe the rationale and design of two ongoing 52-week phase 3 trials (SYGMA1 [NCT02149199, registered on 16 May 2014] and SYGMA2 [NCT02224157, registered on 19 August 2014]) that are comparing as-needed budesonide/formoterol with either maintenance budesonide plus as-needed terbutaline, or as-needed terbutaline alone. Asthma symptom control [2], as assessed by well-controlled asthma weeks, and annual severe exacerbation rate are being investigated as primary endpoints. Secondary endpoints include: average change from baseline in pre-dose forced expiratory volume in 1 s (FEV_1_), Asthma Control Questionnaire (ACQ-5) score, health-related quality of life, asthma symptom control, medication intake measured via the Turbuhaler® Usage Monitor (TUM), the percentage of controller-use days, and adverse events (AEs).

## Methods/design

### Patients

Patients meeting the inclusion and exclusion criteria as detailed in Table 1 were eligible for inclusion in the SYGMA programme. The same inclusion and exclusion criteria apply to both SYGMA1 and SYGMA2.

**Table 1:** Key inclusion and exclusion criteria for participation in the SYGMA programme

Key inclusion criteria	Key exclusion criteria
• Male and female outpatients aged ≥12 years • A documented clinical history of asthma for at least 6 months prior to visit 1, diagnosed according to GINA criteria • Patients in need of GINA (2012) step 2 treatment for the last 30 days before visit 2, i.e. patients with asthma that is either: • Well-controlled on mono-maintenance therapy with either a low, stable dose of an ICS or a LTRA in addition to as-needed use of inhaled short-acting bronchodilator(s) (SABA and/or short-acting anticholinergic agent) • Uncontrolled on inhaled short-acting bronchodilator(s) as needed (SABA and/or short-acting anticholinergic agent) • Lung function tests at visit 2 (according to the ERS guidelines [38]); patients pre-treated with: • Low-dose ICS or LTRA in addition to inhaled short-acting bronchodilator(s) should have pre-bronchodilator FEV_1_ ≥ 80% • An inhaled short-acting bronchodilator only should have pre-bronchodilator FEV_1_ ≥ 60% predicted and post-bronchodilator FEV_1_ ≥ 80% predicted • Reversible airway obstruction at visit 2, defined as an increase in FEV_1_ ≥ 12% and 200 mL relative to baseline, after inhalation of 1 mg terbutaline Turbuhaler®. The test can be repeated at visit 3 in case patients fail at visit 2. If patients fail at both occasions, they can still be included if they have documented historical reversibility within the last 12 months prior to visit 3, with an increase in FEV_1_ ≥ 12% and 200 mL relative to baseline after administration of a rapid-acting β_2_-agonist • For randomisation at visit 3, patients should fulfil the following criteria: • Use of as-needed terbutaline Turbuhaler® due to asthma symptoms on at least 3 separate days during the last week of the run-in period • Ability to use Turbuhaler® correctly and to complete the eDiary correctly. Morning and evening data must be recorded for at least 8 days (any 8) of the last 10 days of the run-in period	• Any asthma worsening requiring change in asthma treatment other than SABA and/or short-acting anticholinergic agent within 30 days prior to visit 1 or during run-in • Use of oral, rectal or parenteral GCS within 30 days and/or depot parenteral GCS within 12 weeks prior to visit 1 • Smoker (current or previous) with a smoking history of ≥10 pack years • Use of any β-blocking agent, including eye drops • Any significant disease or disorder (e.g. cardiovascular, gastrointestinal, hepatic, renal) • Pregnancy, breast-feeding or planned pregnancy • For randomisation at visit 3, patients should not fulfil any of the following criteria: • Use of ≥6 terbutaline Turbuhaler® as-needed inhalations per day, for a certain number of days depending on the actual length of run-in: for ≥2 days out of 14 days; for ≥3 days out of 15–21 days; for ≥4 days out of 22 or more days of run-in

*eDiary* electronic diary, *ERS* European Respiratory Society, *FEV*_*1*_ forced expiratory volume in 1 s, *GCS* glucocorticosteroid, *GINA* Global Initiative for Asthma, *ICS* inhaled corticosteroid, *LTRA* leukotriene receptor antagonist, *SABA* short-acting β_2_-agonist

**Fig. 1:**
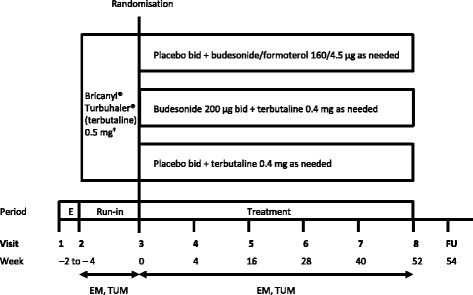
SYGMA1 study design. *bid* twice daily, *E* enrolment, *EM* electronic monitoring twice daily (eDiary), *FU* follow-up phone call, *TUM* Turbuhaler® Usage Monitor, recording use of each blinded study inhalers. ^†^Corresponds to the terbutaline Turbuhaler® 0.4 mg, with regards to the dose delivered

**Fig. 2:**
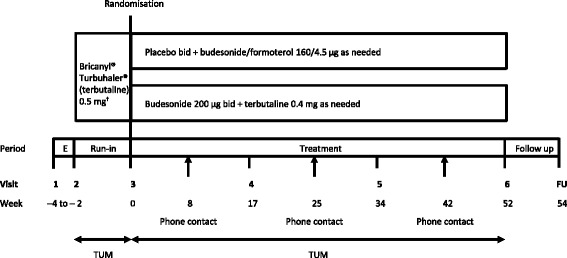
SYGMA2 study design. *bid* twice daily, *E* enrolment, *FU* follow-up phone call, *TUM* Turbuhaler® Usage Monitor, recording use of each blinded study inhalers. ^†^Corresponds to the terbutaline Turbuhaler® 0.4 mg, with regards to the dose delivered

In brief, patients were eligible for inclusion if they were aged 12 years or older at visit 1 (Table 2, Figs. 1 and 2), with a documented clinical diagnosis of asthma according to Global Initiative for Asthma (GINA) criteria [17] for at least 6 months prior to visit 1 and with confirmation of diagnosis by bronchodilator reversibility. Lung function and reversibility tests performed as part of visits 2 and 3 can be used as confirmation of asthma diagnosis if there is no measure of lung function available before visit 1. Patients must have evidence of need for GINA step 2 treatment (low-dose ICS) for the 30 days before visit 2, either by asthma being well-controlled on mono-maintenance therapy with a low stable dose of an ICS or a leukotriene receptor antagonist (LTRA) in addition to as-needed short-acting bronchodilator (SABA and/or short-acting anticholinergic agent) (subgroup 1); or by asthma being uncontrolled on as-needed, inhaled short-acting bronchodilator(s) (subgroup 2). Additional inclusion criteria included baseline pre-bronchodilator FEV_1_ ≥ 80% predicted (for subgroup 1) or ≥60% predicted (for subgroup 2) (Table 1). Exclusion criteria included a recent asthma exacerbation, history of life-threatening asthma requiring intubation, and current or past smokers with a smoking history of at least 10 pack-years.

**Table 2:** Standard Protocol Items: Recommendations for Interventional Trials (SPIRIT) figures for SYGMA1 (A) and SYGMA2 (B)

	Enrolment	Run-in	Randomisation	Treatment						Follow-up
A										
Week		−2 to −4	0	4	16	28	40	52		54
Visit	1	2	3	4	5	6	7	8		Phone
Informed consent	X									
Allocation of enrolment code (IVRS/IWRS)	X									
Demography (date of birth, gender, race)	X									
Inclusion/exclusion criteria	X	X	X							
Medical, surgical history	X									
Asthma history (including history of severe asthma exacerbations)	X									
Smoking history	X									
Patient training in eDiary, Turbuhaler® (inhalation technique), TUM and PEF meter use		X								
ACQ-5 and AQLQ(S) at study site		X	X	Only ACQ-5	X	X	X	X		
SAEs (from visit 1)/AEs (from visit 2)	X	X	X	X	X	X	X	X		X
Weight and height (height only for adolescents at visit 8)		X						X		
Physical examination		X						X		
Pulse and blood pressure		X						X		
Pregnancy test		X								
Adjustment of current asthma medication		X								
Randomisation			X							
Bricanyl® for run-in dispense [d]/return [r]		d	r							
Lung function (FEV_1_, FVC pre and post Bricanyl® administration)		X	X	X	X	X	X	X		
Reversibility test (calculated at visit 2 and if needed, calculated at visit 3 as well)		X	X							
Concomitant medication		X	X	X	X	X	X	X		
Investigational product (dispense [d]/return [r]/check [c])			d	d/r/c	d/r/c	d/r/c	d/r/c	r/c		
Intake of maintenance treatment morning dose			X	X	X	X	X			
Review of PEF, asthma symptoms, night-time awakenings, maintenance and ‘as needed’ IP intake and Turbuhaler® user technique; re-training of patient if needed			X	X	X	X	X	X		
Review of patient’s compliance with eDiary			X	X	X	X	X	X		
Informed consent (qualitative substudy)^a^				X^a^	X^a^	X^a^	X^a^			
B										
Week		−2 to −4	0	8	17	25	34	42	52^b^	54
Visit	1	2	3	Phone		Phone		Phone		
Written informed consent	X									
Allocation of enrolment code	X									
Demography	X									
Inclusion/exclusion criteria	X	X	X							
Medical/surgical history	X									
Asthma history (including exacerbation history)	X									
Smoking history	X									
ACQ-5, AQLQ(S)		X	X		X		X		X	
Health Care resource utilisation questionnaire, EQ-5D-5L			X		X		X		X	
SAE/AEs^c^	X^c^	X^c^	X		X		X		X	X
Weight and height		X							X^d^	
Physical examination		X							X	
Vital signs (pulse and blood pressure)		X							X	
Pregnancy test (if applicable)		X								
Adjustment of current asthma medication		X								
Patient training in how to use Turbuhaler® (inhalation technique) and TUM		X								
Bricanyl® for run- in (dispense [d]/return [r])		d	r							
Randomisation			X							
Lung function (FEV_1_, FVC pre and post Bricanyl® administration)		X	X		X		X		X	
Reversibility test^e^		X	X^e^							
Collection of severe asthma exacerbations				X^f^	X^f^	X^f^	X^f^	X^f^	X^f^	
Concomitant medications		X	X		X		X		X	
Investigational product (dispense [d]/return [r]/check [c])			d		d/r/c		d/r/c		r/c	

Notes:

ACQ-5, Asthma Control Questionnaire 5 questions; AE, adverse event; AQLQ(S), Standardised Asthma Quality of Life Questionnaire; EQ-5D-5L, EuroQol 5-dimensions 5-level; FEV_1_, forced expiratory volume in 1 s; FVC, forced vital capacity; IP, investigational product; IWRS/IVRS, Interactive Web and Voice Response System; PEF, morning peak expiratory flow; SAE, serious adverse event; TUM, Turbuhaler® Usage Monitor, recording use of each blinded study inhalers

^a^Obtaining informed consent of patients into the qualitative substudy is only applicable to the subset of sites selected to participate. Informed consent into the substudy is to be obtained before any interview-related activities. Informed consent can occur at any time at visit 4 (week 4) or later; however, the qualitative patient interview conducted with the patient will occur between week 12 and week 50 for each patient who has elected to participate. The exact time point of the interview will be determined by the contract research organisation

^b^After discontinuation of an investigational drug (ie before visit 6) patients will be followed up according to the original visit schedule including site visits and phone contacts. Only severe asthma exacerbations, AEs and concomitant medications will be collected. If it is not possible for the patient to visit the study site, the visit(s) may be performed via phone

^c^Serious adverse events will be collected from the time of signing informed consent. Adverse events will be collected from visit 2

^d^Height only for adolescents

^e^Reversibility test will be performed at visit 2. The test can be repeated at visit 3 in case the patients fail to meet the inclusion criterion at visit 2

^f^Severe asthma exacerbations will be collected from visit 3 through the entire study

In both studies, patients who fulfilled all the inclusion and none of the exclusion criteria at visit 2 entered a 2–4-week run-in period in order to demonstrate the appropriateness of candidates prior to randomisation (Figs. 1 and 2). Patients were to stop any prescribed asthma medication (including SABA and maintenance treatment with ICS or LTRA) that was being used at the time of study entry, and all patients were to receive as-needed Bricanyl® Turbuhaler® 0.5 mg during run-in (which corresponds to the blinded terbutaline Turbuhaler® 0.4 mg, with regards to the dose delivered). At visit 3, patients will be randomised if they required as-needed terbutaline for the relief of asthma symptoms on at least three separate days during the last week of the run-in period; however, patients will be excluded if, during run-in, they used as-needed terbutaline six or more times per day on: at least 2 days out of 14 days; at least 3 days out of 15–21 days; at least 4 days out of 22 or more days (depending on the duration of run-in). All study medication, including terbutaline during run-in, will be delivered via Turbuhaler® and recorded electronically using the TUM [18].

The study is being performed in accordance with the Declaration of Helsinki and Good Clinical Practice. The protocols for the SYGMA 1 and SYGMA 2 trials have been approved by the oversight authorities in the countries detailed in Additional file 1: Table S1. All patients were required to provide written informed consent before participating in the programme, and recruitment was not to begin in an individual site before all local approvals had been obtained.

### SYGMA1 study design

Target recruitment for SYGMA1 is 3750 patients from approximately 18 countries. These patients will be randomised to either placebo twice daily (bid) plus as-needed budesonide/formoterol 160/4.5 μg, placebo bid plus as-needed terbutaline 0.4 mg, or budesonide 200 μg bid plus as-needed terbutaline 0.4 mg (Fig. 1). Additional short-term (2 − 4 weeks) treatment with open-label inhaled budesonide 200 μg bid is allowed for patients experiencing moderate or severe asthma exacerbations or having long-term poor asthma control. After this time, the patient should be evaluated and ‘stepped down’ to blinded study treatment if possible. If patients require additional budesonide treatment on two separate occasions, either for a moderate exacerbation or for long-term poorly-controlled asthma, the investigator should consider continuing with additional budesonide for the rest of the study. Weeks with open-label budesonide use are not defined as well-controlled asthma weeks. Patients will be instructed to contact the investigator if using more than 12 inhalations/day of as-needed budesonide/formoterol or terbutaline, or if they feel that they are in need of medical assistance. An electronic diary (eDiary) will also alert patients to contact their study physician if symptoms are increasing, and/or if their lung function deteriorates as measured by morning peak expiratory flow (PEF). In addition, all patients will be closely monitored during the course of the study with treatment visits taking place at 4, 16, 28, 40, and 52 weeks after randomisation (Table 2a).

### SYGMA2 study design

Target recruitment for SYGMA2 is 4114 patients from approximately 25 countries. These patients will be randomised to either placebo bid plus as-needed budesonide/formoterol 160/4.5 μg or budesonide 200 μg bid plus as-needed terbutaline 0.4 mg (Fig. 2). Patients will be instructed to contact the investigator if using more than 12 inhalations/day of as-needed budesonide/formoterol or terbutaline, or if they feel that they are in need of medical assistance.

Treatment visits will take place at 17, 34, and 52 weeks after randomisation. Between site visits, study staff will contact patients by phone to record additional asthma treatment and hospitalisation/emergency treatment due to asthma (including severe asthma exacerbation data; Table 2b).

### Efficacy assessments

Details of the primary and secondary efficacy endpoints included in the SYGMA1 and SYGMA2 studies are presented in Table 3. In SYGMA1, the primary objective is to demonstrate that as-needed budesonide/formoterol is superior to as-needed terbutaline in terms of asthma control as measured by well-controlled asthma weeks [19], achieved when two or more of the following criteria are fulfilled: no more than 2 days with a daily asthma symptom score >1; no more than 2 days of as-needed medication use, up to a maximum of four occasions per week (multiple occasions per day are regarded as separate occasions); morning PEF ≥80% predicted every day. Both of the following criteria must also be fulfilled: no night-time awakenings due to asthma; no additional inhaled and/or systemic corticosteroid treatment due to asthma [19]. A secondary objective is to establish the noninferiority of as-needed budesonide/formoterol versus maintenance budesonide plus as-needed terbutaline using the same outcome measure. The primary objective of SYGMA2 is to demonstrate that as-needed budesonide/formoterol is noninferior to twice-daily budesonide plus as-needed terbutaline at reducing the annual severe asthma exacerbation rate (Table 3). Secondary efficacy and safety outcome measures are shown in Table 3.

**Table 3:** Endpoints of the SYGMA programme

	SYGMA1		SYGMA2	
	Comparator	Outcome measure	Comparator	Outcome measure
Primary endpoints	As-needed budesonide/formoterol vs as-needed terbutaline	• Evaluation of asthma control as measured by well-controlled asthma weeks	As-needed budesonide/formoterol vs budesonide bid plus as-needed terbutaline	• Annual severe asthma exacerbation rate
Secondary efficacy endpoints	As-needed budesonide/formoterol vs budesonide bid plus as-needed terbutaline	• Evaluation of asthma control as measured by well-controlled asthma weeks	As-needed budesonide/formoterol vs budesonide bid plus as-needed terbutaline	• Time to first severe asthma exacerbation • Average change from baseline in pre-dose FEV_1_ • Time to study specific asthma-related discontinuation • Average change from baseline in number of inhalations of as-needed medication • Change from baseline in percent of as-needed free days • Percentage of controller use days • Average change from baseline in ACQ-5 score and responders based on MID • Average change from baseline in AQLQ score • Total ICS load and number of days with systemic corticosteroid treatment
	As-needed budesonide/formoterol vs as-needed terbutaline or budesonide bid plus as-needed terbutaline	• Time to first severe asthma exacerbation • Time to first moderate or severe asthma exacerbation • Average change from baseline in pre-dose FEV_1_ • Average change from baseline in morning and evening PEF • Average change from baseline in number of inhalations of as-needed medication • Average change from baseline in symptom score • Percentage of night-time awakenings due to asthma • Percentage of symptom-free days • Percentage of as-needed free days • Percentage of asthma control days • Percentage of controller use days • Time to asthma-related discontinuation • Poorly-controlled asthma weeks • Time to additional steroids for asthma • Average change from baseline in ACQ-5 score and responders based on MID • Average change from baseline in AQLQ score • Total ICS load and number of days with systemic corticosteroid treatment		
Safety endpoints	As-needed budesonide/formoterol vs as-needed terbutaline or budesonide bid plus as-needed terbutaline	• Adverse events (nature, incidence and severity) • Pulse, blood pressure and physical examination	As-needed budesonide/formoterol vs budesonide bid plus as-needed terbutaline	• Adverse events (nature, incidence and severity) • Pulse, blood pressure and physical examination
Exploratory endpoints	N/A	• Coded transcriptions of patient interviews	As-needed budesonide/formoterol vs budesonide bid plus as-needed terbutaline	• EuroQol 5-dimensional 5-level questionnaire • Health Economics Questionnaire for resource utilisation

*ACQ-5* Asthma Control Questionnaire 5-item version, *AQLQ* Asthma Quality of Life Questionnaire standard version, *bid* twice daily, *FEV*_*1*_ forced expiratory volume in 1 s, *ICS* inhaled corticosteroid, *MID* minimal important difference, *PEF* peak expiratory flow

Daily asthma symptom score = the sum of the morning and evening symptom score

In both SYGMA1 and SYGMA2, a severe asthma exacerbation is defined using American Thoracic Society/European Respiratory Society (ATS/ERS) Task Force criteria [20] as worsening of asthma that is associated with a medical intervention, requiring either the use of systemic corticosteroids for at least 3 days (or an injection of depot corticosteroids), or inpatient hospitalisation or an emergency department (ED) visit (or other urgent, unscheduled health care visit) due to asthma that required systemic corticosteroids. Moderate exacerbations will also be assessed in SYGMA1, and are defined as a deterioration of asthma requiring a change in treatment, i.e. initiation of open-label ICS, to avoid progression to a severe exacerbation. Spirometry assessments in both studies will be performed according to ATS/ERS guidelines [21] on the day of study visits (SYGMA1: visits 2–8; SYGMA2: visits 2–6).

In SYGMA1 only, patients will complete an eDiary twice daily for PEF, asthma symptoms and night-time awakenings due to asthma symptoms. A symptom-free day is defined as a day and night with no asthma symptoms, and a night with no awakenings due to asthma symptoms. Similarly, an asthma-control day is defined as the fulfilment of all of the following criteria: a day and night with no asthma symptoms; a night with no awakenings due to asthma symptoms; a day and night with no as-needed medication use. Poorly-controlled asthma weeks, a secondary variable in SYGMA1 only, are defined as the documentation of one of the following conditions: at least two consecutive days with night-time awakening due to asthma symptoms; as-needed medication use for symptom relief on at least three occasions per day for at least two consecutive days; additional systemic corticosteroids required for a severe asthma exacerbation. In SYGMA1, ACQ-5 and Standardised Asthma Quality of Life Questionnaires (AQLQ) will be self-administered within the eDiary at scheduled visits. In SYGMA2, ACQ-5 and AQLQ questionnaires will be self-administered using the electronic Patient Reported Outcome (ePRO) device at scheduled visits.

Use of as-needed or randomised maintenance treatment, and terbutaline use during run-in, will be recorded using the TUM (SmartTurbo™, Adherium, New Zealand) in both studies. The TUM is a validated electronic data logger designed to be attached to a Turbuhaler® [18, 22]. The TUM contains an electronic clock that logs the date and time when the Turbuhaler® base grip is rotated back and forth. Use of open-label budesonide will be recorded in the electronic Case Report Form (eCRF). Patients will receive appropriate training for all devices (eDiary, PEF meter, TUM, ePRO device) at visit 2.

### Safety assessments

Safety assessments included in both studies are detailed in Table 3. AEs will be recorded from visit 2, throughout the treatment and follow-up periods, until the last phone follow-up, or last contact. Serious AEs (SAEs) will be recorded from the time of informed consent. All AEs will be recorded in the eCRF. Physical examination, blood pressure, and pulse rate measurements will be performed before run-in (visit 2) and at the end of treatment (SYGMA1: visit 8; SYGMA2: visit 6). Follow-up phone contact for AEs will be performed 2 weeks after completion of study treatment. An independent Adjudication Committee will review any fatal events occurring during the SYGMA studies to determine whether these events were asthma-related.

### Exploratory assessments

In SYGMA1, a qualitative substudy is being conducted in a small subset of patients. The exploratory objective of this substudy is to further evaluate when and why patients use the study medications, using a qualitative interview approach [23]. In SYGMA2, information on health care resource utilisation as well as health status (EuroQol 5-dimensions 5-level health survey: EQ-5D-5L) will be collected at randomisation and at each treatment visit to enable the cost-effectiveness of the interventions to be assessed. Patients will be asked about ambulatory-setting or home consultations with specialists, primary care physicians or other health care professionals, and phone consultations with physicians or nurses as well as health care resource use in terms of: ambulance services; ED visits; hospital admissions including intensive care. Patients in paid employment and education will be asked how much time they have missed due to their asthma. The EQ-5D-5 L will assess mobility, self-care, usual activity, pain/discomfort, and anxiety/depression, and will be self-administered using the paper version.

### Discontinuations

Study-specific criteria for discontinuations in SYGMA1 are: a severe asthma exacerbation with a duration of more than 3 weeks; two severe asthma exacerbations within a period of 3 months; or three severe asthma exacerbations in total during the study. Study-specific criteria for discontinuations in SYGMA2 are: a severe asthma exacerbation with duration of more than 3 weeks; three severe asthma exacerbations within a period of 6 months.

### Sample size estimates

In SYGMA1, 3750 patients (625 patients/treatment group/pre-study treatment group) are required to give greater than 95% power to detect superiority of as-needed budesonide/formoterol compared with as-needed terbutaline and 90% power to establish noninferiority of as-needed budesonide/formoterol compared with budesonide plus as-needed terbutaline for well-controlled asthma weeks, with a pre-defined noninferiority limit of 0.8, i.e. the lower 95% confidence interval (CI) of the odds ratio for budesonide/formoterol versus budesonide plus terbutaline is ≥0.8. Assuming that equal numbers of patients are recruited to each of the subgroups (stratified by pre-study treatment), this sample size also gives 80% power to detect a difference between as-needed budesonide/formoterol and as-needed terbutaline, and 80% power to establish noninferiority of as-needed budesonide/formoterol compared with budesonide plus as-needed terbutaline, with a pre-defined noninferiority limit of 0.78.

SYGMA2 was initially powered to demonstrate superiority of as-needed budesonide/formoterol compared with budesonide plus as-needed terbutaline as measured by the annualised severe exacerbation rate; 4114 patients (2057 patients/treatment group) were estimated to be required to achieve 90% power to detect a difference in annualised severe asthma exacerbation rate between treatments, assuming an exacerbation rate of 0.16 per year among patients treated with budesonide, with a 25% reduction in risk for patients receiving as-needed budesonide/formoterol. To account for uncertainty over the assumed exacerbation rates as well as the dispersion parameter, the overall exacerbation rate was planned to be monitored during the study in a blinded fashion, allowing for an increase of the sample size by a maximum of 50%. A blinded sample size review of SYGMA 2, performed according to the study protocol and prior to enrolling the last patient, indicated that there would be adequate power to test a noninferiority hypothesis based on the design and assumptions specified in the protocol. The SYGMA 2 protocol was amended to include a noninferiority test as the primary analysis, using a pre-defined noninferiority limit of 1.2.

### Statistical analyses

The primary outcome measure in SYGMA1, well-controlled asthma weeks (as-needed budesonide/formoterol versus as-needed terbutaline (superiority) and as-needed budesonide/formoterol versus maintenance budesonide plus as-needed terbutaline (noninferiority)), will be analysed by a repeated measures logistic regression model with: treatment, pre-study treatment, and region as fixed effects; study week as a categorical time variable; and patient as a random effect. The statistical inference will be based on the estimated odds ratio and corresponding 95% CI averaged over the whole randomised treatment period.

In SYGMA2, the primary outcome measure of severe exacerbation rate (as-needed budesonide/formoterol versus maintenance budesonide plus as-needed terbutaline (noninferiority)) will be analysed by a negative binomial regression model with treatment, pre-study treatment group, and region as factors. Annual severe exacerbation rates will be estimated and treatment effect will be expressed as the rate ratio and corresponding one-sided noninferiority interval and two-sided 95% CI. If noninferiority is achieved, then a test for superiority of as needed budesonide/formoterol versus maintenance budesonide will be performed.

The following secondary efficacy endpoints will be assessed in SYGMA1 only. The moderate-to-severe and severe asthma exacerbation rate will be analysed by a negative binomial regression model with treatment, pre-study treatment, and region as factors, and presented as rate ratios and 95% CIs. Time to first moderate-to-severe exacerbation and time to administration of additional steroids will be analysed by a Cox proportional hazards model with treatment, pre-study treatment group, and region as factors; hazard ratios (HRs) and 95% CIs will be estimated. Change in eDiary variables from run-in to the mean value of available data during treatment will be analysed by analysis of covariance (ANCOVA) with treatment, pre-study treatment, and region as factors, and mean values during run-in as a continuous covariate. Least squared means (LSMs) by treatment and differences in LSMs between treatments will be estimated, along with corresponding 95% CIs. Poorly-controlled asthma weeks will be analysed in the same manner as the primary outcome measure, well-controlled asthma weeks.

The remaining secondary efficacy endpoints will be analysed in both SYGMA1 and SYGMA2. Time to first severe asthma exacerbation and time to discontinuation due to asthma-related events will be analysed by a Cox proportional hazards model with treatment, pre-study treatment group, and region as factors; HRs and 95% CIs will be estimated. The treatment effect and 95% CI for average change from baseline in pre-dose FEV_1_, ACQ-5, and AQLQ will be analysed using a mixed-model repeated measures analysis, with the analysis including terms for treatment, pre-study treatment group, region, visit, and treatment by visit. FEV_1_ data will be analysed with baseline FEV_1_ included as a covariate. For ACQ-5 and AQLQ, change from baseline to the end of treatment will also be analysed by ANCOVA with treatment, pre-study treatment group, and region as factors and baseline as a continuous variable. Responder variables, based on minimal important difference for ACQ-5 and AQLQ, will be analysed using a logistic regression model with treatment, region, and pre-study treatment as factors, and baseline as a covariate. From the logistic regression model, treatment effects will be estimated by odds ratio and its corresponding 95% CI. The percentage of controller-use days and as-needed use will be analysed by ANCOVA with treatment, pre-study treatment group, and region as factors; LSMs by treatment and differences in LSMs between treatments will be estimated along with corresponding 95% CIs. In SYGMA1, sensitivity analyses will be performed to explore the impact of the individual components of well-controlled asthma weeks, paying specific attention to the as-needed component. In SYGMA2, a sensitivity analysis for the primary variable will include all data for patients who discontinue study medication but remain in the study.

For both studies, AEs will be listed for each patient and summarised by means of count summaries by System Organ Class and Preferred Term assigned to the event. Other safety variables will be summarised as appropriate. The exploratory endpoints (resource utilisation and health-related quality of life) will be reported descriptively as part of SYGMA2.

In order to assess the consistency of the treatment effect in the two subgroups (defined by pre-study treatment), a pre-study treatment × treatment interaction term will be included in the models for the primary variables in SYGMA1 and SYGMA2. Several other subgroup analyses are also planned, including age, gender, severe exacerbation history in the 12 months prior to screening, baseline symptom history (ACQ), time since asthma diagnosis, smoking history, region, pre-bronchodilator FEV_1,_ and SABA use during run-in.

## Discussion

Both clinicians and patients may underestimate the risks and burden associated with mild asthma. Indeed, despite the proven efficacy of ICS maintenance therapy in asthma, there remains an unmet medical need due to over-reliance on SABA reliever medication and poor adherence to prescribed ICS [24]. Initial reports suggest that the as-needed use of combination ICS/β_2_-agonist with rapid onset of action may have advantages over regular ICS therapy for the treatment of mild asthma [11, 12]. For example, in a 6-month trial of 455 patients with mild asthma, the symptom-driven use of BDP/salbutamol was as effective for asthma exacerbations and morning PEF as maintenance BDP or BDP/salbutamol plus as-needed salbutamol [11], and significantly better than as-needed salbutamol.

Large randomised controlled trials investigating the as-needed use of different ICS/LABA combinations, taken in response to symptoms, as an alternative to current step 2 treatment in patients with mild asthma are currently lacking and are an important research priority [15, 16]. SYGMA1 and SYGMA2 are the first major randomised controlled trials to assess the as-needed use of budesonide/formoterol in patients with mild asthma. For SYGMA2, severe exacerbations are the primary outcome measure. However, for SYGMA1, well-controlled asthma weeks was chosen as the primary outcome measure due to its relevance to all patients. Consequently, the primary outcomes of the SYGMA studies address both aspects of the goals of asthma management, as defined by GINA and other treatment guidelines, which are to achieve good symptom control and to minimise the future risk of exacerbations.

Well-controlled asthma weeks has previously been used in several studies as a composite measure of asthma control [19, 25, 26], but, to the best of our knowledge, this outcome measure has never been used in studies that include electronic monitoring of reliever use. Well-controlled asthma weeks takes into account symptoms, night-time awakenings, lung function, and as-needed reliever medication use, and corresponds to the ‘symptom control’ component in current guidelines, and excludes weeks with additional corticosteroid use or with night-time awakenings. However, the definition of well-controlled asthma weeks is based on current guidelines, where ‘as needed’ medication is usually a short-acting bronchodilator that does not contain an anti-inflammatory component, and so it may not be applicable to patients receiving as-needed budesonide/formoterol. Consequently, a sensitivity analysis will be performed that excludes the as-needed use of study medication from the definition of well-controlled asthma weeks. In addition, although patients are known to over-report their preventer use, they often under-report their reliever use [27]; as a result, there may be fewer well-controlled asthma weeks with reliever use recorded electronically than if this element had been based on self-report. It should also be noted that the TUM is electronically recording the turning of the Turbuhaler® grip, and not the actual process of inhalation, which may lead to over reporting of medication use.

A strength of the SYGMA studies is their long duration (52 weeks), which will allow reliable assessment of exacerbations. Reducing future risk by the prevention of asthma exacerbations is a key goal of asthma management [2] as exacerbations constitute the greatest risk to patients, are a cause of anxiety to patients and their families, and result in the greatest cost to the health care system [20]. There is some uncertainty over the frequency of severe exacerbations in mild asthma, with estimates ranging from 0.12 to 0.77 episodes per patient-year [1, 3, 28]. It has been suggested that severe exacerbations in mild asthma represent 30–40% of all asthma exacerbations that require an emergency consultation [1]. Twelve months is regarded as the minimum period for evaluation of annualised exacerbation rates [29]; this is particularly important in mild asthma, in which exacerbations are relatively rare events [30]. The designs of the SYGMA studies utilise clinic visits (SYGMA1 and SYGMA2), eDiary alerts (SYGMA1 only), and additional phone contacts (SYGMA2 only) to ensure that asthma exacerbation events are correctly collected.

The 52-week duration and use of an electronic monitoring device (TUM) on all blinded study inhalers will also allow evaluation of usage of the randomised maintenance and as-needed medications over all seasons of a year. Patients with mild asthma may be willing to accept mild symptoms and have poor adherence to maintenance ICS [31]. It is, therefore, hoped that the as-needed approach will better match patient behaviour and help to overcome the problems associated with poor adherence. Patients will be aware that inhaler use is being recorded, which may temporarily improve adherence with maintenance treatment; however, this is likely to occur only for a short period of time [32].

A number of other considerations have contributed to the design of the studies. For example, a 2–4-week run-in period has been included in both studies to allow baseline data to be collected and to ensure that patients are in need of GINA step 2 treatment. Furthermore, in SYGMA1 only, patient recruitment will be balanced to allow stratification based on pre-study treatment, i.e. asthma that is uncontrolled on as-needed short-acting bronchodilator or asthma that is well-controlled on mono-maintenance with either a low-dose ICS or LTRA plus as-needed short-acting bronchodilator. Health economic data will be collected in SYGMA2, to allow comparison of the cost-effectiveness of the different regimens under different pricing structures.

Data for the use of as-needed budesonide/formoterol compared with as-needed SABA in a similar patient population and in studies with a similar design to SYGMA are unavailable; as such, the SYGMA1 study includes a ‘pseudo’ placebo arm that allows the assessment of the superiority of as-needed budesonide/formoterol compared with as-needed terbutaline. However, patients in the ‘pseudo’ placebo arm may experience a deterioration in asthma control, and so it is important to ensure that there is a mechanism by which they receive rapid medical attention whenever necessary. Consequently, triggers in the eDiary generate alerts that warn patients of worsening asthma, prompting them to contact the investigator who may prescribe additional inhaled and/or systemic corticosteroid treatment to patients experiencing an exacerbation or with poor long-term asthma control. In addition, patients will be instructed to contact the investigator at any time should they require medical assistance or if they use more than 12 as-needed inhalations per day. The SYGMA2 study is designed to support the SYGMA1 regulatory study but reduce the burden of study requirements, e.g. fewer scheduled visits and absence of the eDiary/patient reminders. However, because of the regulatory requirements for a double-blind, placebo-controlled study, all participants will be required to take a regular maintenance inhaler containing either placebo or budesonide to ensure blinding of the treatment groups. Consequently, additional studies with a pragmatic, open-label design will be needed to evaluate natural patient behaviour with an as-needed regimen, so that the results can be generalised to clinical practice, and two such studies (Universal Trial Numbers: U1111-1170-2118; U1111-1174-2273) are underway [33].

In SYGMA 2, daily low-dose budesonide (plus as-needed terbutaline) was selected as the active comparator treatment. The use of regular daily low-dose budesonide is well-established, having been shown to be highly effective in reducing the risk of asthma-related exacerbations, including in patients with mild persistent asthma (as demonstrated in the START study) [3]. Furthermore, additional evidence has recently emerged confirming the appropriateness of low-dose budesonide maintenance therapy for patients with mild asthma and less frequent symptoms [34, 35].

Thus, the availability of an as-needed ICS/LABA single inhaler that could provide comparable control to current standard-of-care treatment with low-dose daily ICS plus as-needed medication would represent a breakthrough in the treatment of mild asthma, providing patients and clinicians with an alternative more convenient option with the potential for improved adherence. The blinded sample size review conducted during SYGMA 2 demonstrated adequate power to test a noninferiority hypothesis based on the design and assumptions specified in the study protocol. In view of this, the decision was taken to change the primary objective for SYGMA 2 to assess whether budesonide/formoterol as needed will be noninferior to budesonide given as regular maintenance with regard to the annual severe exacerbation rate. To maintain the validity of the primary analysis, the protocol was amended prior to trial completion and unblinding, and the noninferiority margin relative to the control arm was pre-specified [36, 37].While a noninferiority margin for exacerbation reduction in patients with mild asthma has not been previously defined, a margin of 20% was considered to be an appropriate choice because a difference between treatments smaller than this would be judged to be of questionable clinical relevance in this population. If the noninferiority criteria are met in SYGMA 2, superiority of as-needed budesonide/formoterol versus budesonide will also be assessed. The benefit/risk will also be assessed in the context of other endpoints evaluating asthma control as well as steroid load, the pattern of ‘as needed use’ and controller use days.

In conclusion, the SYGMA programme should help to determine the efficacy and safety of as-needed budesonide/formoterol combination therapy in mild asthma, as an alternative to regular low-dose ICS treatment. Patient recruitment is completed and completion of the phase 3 studies is planned in 2017.

## Trial status

The first patients were recruited to SYGMA1 and SYGMA2 in July 2014 and November 2014, respectively. Recruitment to both trials is completed (June 2016).

## Additional files

Additional file 1: Table S1. Oversight Authorities for SYGMA1 and SYGMA2. (DOCX 20 kb)
Additional file 2: SPIRIT Checklist for SYGMA1 and SYGMA2. (DOCX 52 kb)

## Abbreviations

ACQ-5: Asthma Control Questionnaire 5-item version
AE: Adverse event
ANCOVA: Analysis of covariance
AQLQ: Standardised Asthma Quality of Life Questionnaire
ATS: American Thoracic Society
bid: Twice daily
BDP: Beclometasone dipropionate
CI: Confidence interval
eCRF: Electronic Case Report Form
ED: Emergency department
eDiary: Electronic diary
ePRO: Electronic Patient Reported Outcome
ERS: European Respiratory Society
FeNO: Fractional exhaled nitric oxide
FEV_1_: Pre-dose forced expiratory volume in 1 s
GCS: Glucocorticosteroid
GINA: Global Initiative for Asthma
HR: Hazard ratio
ICS: Inhaled corticosteroid
LABA: Long-acting β_2_-agonist
LSM: Least squared mean
LTRA: Leukotriene receptor antagonist
PEF: Peak expiratory flow
SABA: Short-acting β_2_-agonist
SAE: Serious adverse event
SYGMA: SYmbicort Given as needed in Mild Asthma
TUM: Turbuhaler® Usage Monitor

## References

[1] Dusser D, Montani D, Chanez P, de Blic J, Delacourt C, Deschildre A (2007). Mild asthma: an expert review on epidemiology, clinical characteristics and treatment recommendations. Allergy.

[2] Global Strategy for Asthma Management and Prevention. Updated 2015. Global Initiative for Asthma. 2015. http://ginasthma.org/wp-content/uploads/2016/01/GINA_Report_2015_Aug11-1.pdf. Accessed 13 Oct 2015.

[3] Pauwels RA, Pedersen S, Busse WW, Tan WC, Chen YZ, Ohlsson SV (2003). Early intervention with budesonide in mild persistent asthma: a randomised, double-blind trial. Lancet.

[4] Patel M, Pilcher J, Pritchard A, Perrin K, Travers J, Shaw D (2013). Efficacy and safety of maintenance and reliever combination budesonide-formoterol inhaler in patients with asthma at risk of severe exacerbations: a randomised controlled trial. Lancet Respir Med.

[5] Barnes CB, Ulrik CS (2015). Asthma and adherence to inhaled corticosteroids: current status and future perspectives. Respir Care.

[6] Suissa S, Ernst P, Benayoun S, Baltzan M, Cai B (2000). Low-dose inhaled corticosteroids and the prevention of death from asthma. N Engl J Med.

[7] Why asthma still kills: The National Review of Asthma Deaths (NRAD). Royal College of Physicians. 2014. https://www.rcplondon.ac.uk/file/868/download?token=3wikiuFg. Accessed 13 Oct 2015.

[8] Suissa S, Ernst P, Kezouh A (2002). Regular use of inhaled corticosteroids and the long term prevention of hospitalisation for asthma. Thorax.

[9] Williams LK, Peterson EL, Wells K, Ahmedani BK, Kumar R, Burchard EG (2011). Quantifying the proportion of severe asthma exacerbations attributable to inhaled corticosteroid nonadherence. J Allergy Clin Immunol..

[10] Rabe KF, Adachi M, Lai CK, Soriano JB, Vermeire PA, Weiss KB (2004). Worldwide severity and control of asthma in children and adults: the global asthma insights and reality surveys. J Allergy Clin Immunol.

[11] Papi A, Canonica GW, Maestrelli P, Paggiaro P, Olivieri D, Pozzi E (2007). Rescue use of beclomethasone and albuterol in a single inhaler for mild asthma. N Engl J Med.

[12] Calhoun WJ, Ameredes BT, King TS, Icitovic N, Bleecker ER, Castro M (2012). Comparison of physician-, biomarker-, and symptom-based strategies for adjustment of inhaled corticosteroid therapy in adults with asthma: the BASALT randomized controlled trial. JAMA.

[13] Haahtela T, Tamminen K, Malmberg LP, Zetterstrom O, Karjalainen J, Yla-Outinen H (2006). Formoterol as needed with or without budesonide in patients with intermittent asthma and raised NO levels in exhaled air: a SOMA study. Eur Respir J.

[14] Rabe KF, Atienza T, Magyar P, Larsson P, Jorup C, Lalloo UG (2006). Effect of budesonide in combination with formoterol for reliever therapy in asthma exacerbations: a randomised controlled, double-blind study. Lancet.

[15] Papi A, Caramori G, Adcock IM, Barnes PJ (2009). Rescue treatment in asthma. More than as-needed bronchodilation. Chest.

[16] Beasley R, Weatherall M, Shirtcliffe P, Hancox R, Reddel HK (2014). Combination corticosteroid/beta-agonist inhaler as reliever therapy: a solution for intermittent and mild asthma?. J Allergy Clin Immunol.

[17] Global Strategy for Asthma Management and Prevention. Updated 2012. Global Initiative for Asthma. 2012. http://www.ginasthma.org/local/uploads/files/GINA_Report_March13_1.pdf. Accessed 13 Oct 2015.

[18] Gradinarsky L, Lööf T (2014). Inhalation adherence monitoring using smart electronic add-on device. MobiHealth: 4th International Conference on Wireless Mobile Communication and Healthcare – ‘Transforming healthcare through innovations in mobile and wireless technologies’.

[19] Bateman ED, Boushey HA, Bousquet J, Busse WW, Clark TJ, Pauwels RA (2004). Can guideline-defined asthma control be achieved? The Gaining Optimal Asthma ControL study. Am J Respir Crit Care Med.

[20] Reddel HK, Taylor DR, Bateman ED, Boulet LP, Boushey HA, Busse WW (2009). An official American Thoracic Society/European Respiratory Society statement: asthma control and exacerbations: standardizing endpoints for clinical asthma trials and clinical practice. Am J Respir Crit Care Med.

[21] Miller MR, Hankinson J, Brusasco V, Burgos F, Casaburi R, Coates A (2005). Standardisation of spirometry. Eur Respir J.

[22] Pilcher J, Shirtcliffe P, Patel M, McKinstry S, Cripps T, Weatherall M (2015). Three-month validation of a turbuhaler electronic monitoring device: implications for asthma clinical trial use. BMJ Open Resp Res..

[23] Barbour RS (2000). The role of qualitative research in broadening the ‘evidence base’ for clinical practice. J Eval Clin Pract.

[24] O’Byrne P, Cuddy L, Taylor DW, Birch S, Morris J, Syrotuik J (1996). Efficacy and cost benefit of inhaled corticosteroids in patients considered to have mild asthma in primary care practice. Can Respir J.

[25] NCT00463866. Local Phase 4 Pan-European SMART Study. 2015. https://www.clinicaltrials.gov/ct2/show/NCT00463866. Accessed 16 June 2015.

[26] NCT00315744. Viapaed Study in children and adolescents with asthma. 2015. https://www.clinicaltrials.gov/ct2/show/NCT00315744. Accessed 16 June 2015.

[27] Patel M, Perrin K, Pritchard A, Williams M, Wijesinghe M, Weatherall M (2013). Accuracy of patient self-report as a measure of inhaled asthma medication use. Respirology.

[28] O’Byrne PM, Barnes PJ, Rodriguez-Roisin R, Runnerstrom E, Sandstrom T, Svensson K (2001). Low dose inhaled budesonide and formoterol in mild persistent asthma: the OPTIMA randomized trial. Am J Respir Crit Care Med.

[29] Fuhlbrigge A, Peden D, Apter AJ, Boushey HA, Camargo CA, Gern J (2012). Asthma outcomes: exacerbations. J Allergy Clin Immunol.

[30] de Vries F, Setakis E, Zhang B, van Staa TP (2010). Long-acting {beta}2-agonists in adult asthma and the pattern of risk of death and severe asthma outcomes: a study using the GPRD. Eur Respir J.

[31] Shahidi N, Fitzgerald JM (2010). Current recommendations for the treatment of mild asthma. J Asthma Allergy..

[32] Foster JM, Smith L, Bosnic-Anticevich SZ, Usherwood T, Sawyer SM, Rand CS (2012). Identifying patient-specific beliefs and behaviours for conversations about adherence in asthma. Intern Med J.

[33] Beasley R, Pavord I, Papi A, Reddel HK, Harrison T, Marks G et al. Description of randomised controlled trial of ICS/LABA reliever therapy in mild asthma. Eur Respir J. 2015. In press. 10.1183/13993003.01692-2015 26846834

[34] Reddel HK, Busse WW, Pedersen S, Tan WC, Chen Y-Z, Jorup C, et al. Should recommendations about starting inhaled corticosteroid treatment for mild asthma be based on symptom frequency: a post-hoc efficacy analysis of the START study. Lancet. 2016. doi:10.1016/S0140-6736(16)31399-X. 10.1016/S0140-6736(16)31399-X 27912982

[35] Global Strategy for Asthma Management and Prevention. Updated 2016. Global Initiative for Asthma. 2016. http://ginasthma.org/2016-gina-report-global-strategy-for-asthma-management-and-prevention/. Accessed 7 Oct 2016

[36] CPMP. Committee for Proprietary Medicinal Products. London; 2000. http://www.ema.europa.eu/docs/en_GB/document_library/Scientific_guideline/2009/09/WC500003658.pdf. Accessed 7 Oct 2016.

[37] Pocock SJ, Stone GW (2016). The primary outcome fails—What next?. N Engl J Med.

[38] Quanjer PH, Stanojevic S, Cole TJ, Baur X, Hall GL, Culver BH (2012). Multi-ethnic reference values for spirometry for the 3-95-yr age range: the global lung function 2012 equations. Eur Respir J.

