# Supplementary material for: The SYGMA programme of phase 3 trials to evaluate the efficacy and safety of budesonide/formoterol given ‘as needed’ in mild asthma: study protocols for two randomised controlled trials
Source: Trials. 2017 Jan 10;18:12. doi: 10.1186/s13063-016-1731-4 (PMC5223341; doi:10.1186/s13063-016-1731-4)
Supplement: Additional file 1: Table S1. — Oversight Authorities for SYGMA1 and SYGMA2. (DOCX 20 kb) [file 13063_2016_1731_MOESM1_ESM.docx]

**Additional file 1: Table S1**

**Oversight Authorities for SYGMA1**

Australia: Department of Health and Ageing Therapeutic Goods Administration
Australia: Human Research Ethics Committee

Brazil: National Committee of Ethics in Research

Brazil: National Health Surveillance Agency
Bulgaria: Bulgarian Executive Drug Agency
Bulgaria: Ethics Committee for Multicentre Studies

Canada: Health Canada

Chile: Instituto de Salud Pública
China: State Food and Drug Administration

Hungary: National Institute of Pharmacy
Hungary: Research Ethics Medical Committee

Korea: Ministry of Food and Drug Safety
Mexico: Comité Bioético para la Investigación Clínica
Mexico: Comité de Ética en Investigación de la Facultad de Medicina y Hospital Universitario

Mexico: Federal Commission for Protection Against Health Risks
Peru: Instituto Nacional de Salud

Philippines: Bureau of Food and Drugs
Poland: Komisja Bioetyczna Przy Okręgowej Izbie Lekarskiej W Białymstoku

Poland: Office for Registration of Medicinal Products, Medical Devices and Biocidal Products
Romania: National Medicines and Medical Devices Agency

Russia: Ethics Committee of the Ministry Of Health of the Russian Federation

Russia: Ministry of Health of the Russian Federation
South Africa: Medicines Control Council
Ukraine: Central Ethics Committee of the Ministry of Health of Ukraine

Ukraine: State Pharmacological Center – Ministry of Health
United Kingdom: Medicines and Healthcare Products Regulatory Agency
Vietnam: Ministry of Health

**Oversight Authorities for SYGMA2**

Australia: Department of Health and Ageing Therapeutic Goods Administration
Australia: Human Research Ethics Committee
Brazil: National Committee of Ethics in Research

Brazil: National Health Surveillance Agency
Bulgaria: Bulgarian Executive Drug Agency
Bulgaria: Ethics Committee for Multicentre Studies
Chile: Instituto de Salud Pública
Colombia: Ministry of Health / National Institute of Food and Drug Monitoring
Czech Republic: State Institute for Drug Control
France: National Agency for the Safety of Medicines and Health Products
Germany: Federal Institute for Drugs and Medical Devices / Ministry of Health / Paul Erlich Institute sin Langen
Hungary: National Institute of Pharmacy
Hungary: Research Ethics Medical Committee
Mexico: Comité Bioético para la Investigación Clínica
Mexico: Comité de Ética en Investigación de la Facultad de Medicina y Hospital Universitario

Mexico: Federal Commission for Protection Against Health Risks
New Zealand: Medicines and Medical Devices Safety Authority / New Zealand Ministry of Health / PHARMAC
Peru: Instituto Nacional de Salud
Philippines: Bureau of Food and Drugs
Poland: Komisja Bioetyczna przy Wielkopolskiej Izbie Lekarskiej

Poland: Urzad Rejestracji Produktow Leczniczych Wyrobow Medycznych i Produktow Biobojczych
Romania: National Medicines and Medical Devices Agency
Russia: Ethics Committee of the Ministry Of Health of the Russian Federation

Russia: Ministry of Health of the Russian Federation
Saudi Arabia: National Committee for Medical and Bio-ethics, Kin Abdulaziz City for Science and Technology

Saudi Arabia: Saudi Food and Drug Authority Contact
Slovakia: Etika Komisa Urad Kosickeho Samospravneho Kraja

Slovakia: State Institute for Drug Control / Ministry of Health
South Africa: Medicines Control Council
South Korea: Ministry of Food and Drug Safety
Spain: Spanish Agency for Medicines and Health Products
Sweden: Medical Products Agency
Thailand: Ministry of Public Health
Ukraine: Central Ethics Committee of the Ministry of Health of Ukraine
Ukraine: State Pharmacological Center – Ministry of Health
Vietnam: Ministry of Health
